# Sleep apnea predicts cardiovascular death in patients with Marfan syndrome: a cohort study

**DOI:** 10.1007/s13167-022-00291-4

**Published:** 2022-07-29

**Authors:** Nele Gessler, Peter Wohlmuth, Omar Anwar, Eike Sebastian Debus, Christian Eickholt, Melanie A Gunawardene, Samer Hakmi, Kathrin Heitmann, Meike Rybczynski, Helke Schueler, Sara Sheikhzadeh, Eike Tigges, Gunther H Wiest, Stephan Willems, Ekaterina Adam, Yskert von Kodolitsch

**Affiliations:** 1 Department of Cardiology and Internal Intensive Care Medicine, Asklepios Clinic St. Georg, Semmelweis University, Campus Hamburg, Lohmuehlenstrasse 5, 20099 Hamburg, Germany; 2 DZHK (German Center for Cardiovascular Research), Partner Site Hamburg/Kiel/Luebeck, Berlin, Germany; 3 Asklepios Proresearch, Research Institute, Hamburg, Germany; 4 University Heart Center Hamburg Eppendorf, Hamburg, Germany; 5 Emergency Department, Asklepios Clinic St. Georg, Semmelweis University, Campus Hamburg, Hamburg, Germany; 6 Emergency Department, Asklepios Clinic Harburg, Semmelweis University, Campus Hamburg, Hamburg, Germany; 7 Department of Pneumology and Sleep Medicine, Asklepios Clinic Harburg, Semmelweis University, Campus Hamburg, Hamburg, Germany

**Keywords:** Predictive preventive personalized medicine, Marfan syndrome, Sleep apnea, Mortality, Connective tissue deficits, Cardiovascular death, Aortic rupture

## Abstract

**Background:**

Surgical replacement of the aortic root is the only intervention that can prevent aortic dissection and cardiovascular death in Marfan syndrome (MFS). However, in some individuals, MFS also causes sleep apnea. If sleep apnea predicts cardiovascular death, a new target for predictive, preventive, and personalized medicine (PPPM) may emerge for those individuals with MFS who have sleep apnea.

**Methods:**

This is an investigator-initiated study with long-term follow-up data of 105 individuals with MFS. All individuals were screened for sleep apnea regardless of symptoms. Cardiovascular death served as a primary endpoint, and aortic events as a secondary outcome.

**Results:**

Sleep apnea with an apnea–hypopnea index (AHI) > 5/h was observed in 21.0% (22/105) with mild sleep apnea in 13% (14/105) and moderate to severe sleep apnea in 7.6% (8/105). After a median follow-up of 7.76 years (interquartile range: 6.84, 8.41), 10% (10/105) had died, with cardiovascular cause of death in 80% (8/10). After adjusting for age and body mass index (BMI), the AHI score emerged as an independent risk factor for cardiovascular death (hazard ratio 1.712, 95% confidence interval [1.061–2.761], *p* = 0.0276). The secondary outcome of aortic events occurred in 33% (35/105). There was no effect of the AHI score on aortic events after adjusting for age and BMI (hazard ratio 0.965, 95% confidence interval [0.617–1.509]), possibly due to a high number of patients with prior aortic surgery.

**Interpretation:**

Sleep apnea is emerging as an independent predictor of cardiovascular death in MFS. It seems mandatory to screen all individuals with MFS for sleep apnea and to include these individuals, with both MFS and sleep apnea, in further studies to evaluate the impact of preventive measures with regard to cardiovascular death.

**Supplementary Information:**

The online version contains supplementary material available at 10.1007/s13167-022-00291-4.

## Introduction

Marfan syndrome (MFS) is an inherited connective tissue disorder with an incidence of 2–3 per 10,000 individuals, caused by mutations in the *FBN*1 gene [1]. It is a multisystem disorder with manifestations typically involving the cardiovascular, skeletal, and ocular systems, inherited in a dominant manner with 25% of cases being sporadic due to de novo mutations [1]. Individuals with MFS have a high risk for cardiovascular death due to aortic aneurysms and dissections [2]. Before the development of preventive open heart surgical procedures for prophylactic replacement of the aortic root, Marfan patients usually died at a mean age of 32 years [3]. Life expectancy has increased because of elective replacement of the proximal aorta before aortic dissection or rupture develop [4]. Nevertheless, the risk for cardiovascular death remains high, including ventricular arrhythmias and myocardial involvement [5].

The current management of MFS is a standardized approach for all patients with yearly measurements of the aorta in greatest diameter and monitoring of the aortic growth rate in order to establish the appropriate time to intervene surgically [1, 6, 7]. Surgical replacement of the aortic root is the exclusive standard for saving all individuals with MFS from premature aortic dissection and cardiovascular death [4]. However, some individuals with MFS manifest sleep apnea at an unusually young age [8–10]. The relationship between Marfan syndrome and sleep apnea is well known with a reported prevalence of obstructive sleep apnea between 31 and 42.5% in observational studies [8–10]. Craniofacial abnormalities and increased upper airway collapsibility during sleep were suspected as possible causes for sleep apnea [11, 12].

An association between the occurrence of sleep apnea and aortic events was previously suggested [13] but not confirmed [8, 9]. Thus far, no long-term follow-up data are available regarding this topic. Sleep apnea is a well-known risk factor for several cardiovascular diseases, such as hypertension, arrhythmias, and congestive heart failure [14].

Until now, the predictive effect of sleep apnea on cardiovascular mortality has never been investigated in patients with MFS or other heritable thoracic aortic diseases (HTAD). Therefore, we designed this study to determine whether sleep apnea in MFS can provide a new leverage to personalize the risk of aortic dissection and death. If sleep apnea predicts cardiovascular death, a new target for predictive, preventive, and personalized medicine (PPPM) may emerge for individuals with MFS and sleep apnea. Targeted sleep apnea screening of individuals with MFS may pave the way for future personalized preventive treatment.

Hence, the aim of this study was to evaluate the effect of sleep apnea on cardiovascular death and on aortic events in a large cohort of individuals with genetically confirmed MFS.

## Study design and methods

### Study design and population

In this observational study, all adult patients presenting at the MFS outpatient clinic of a tertiary care center were offered screening for sleep apnea regardless of symptoms. These were individuals with previously diagnosed MFS presenting for regular follow-up visits and individuals with suspected MFS undergoing thorough clinical examination and genetic testing, all in accordance with current guidelines [15, 16] or expert recommendations [17]. If the diagnosis of MFS was not confirmed, individuals were excluded from the study, as were patients already on continuous positive airway pressure therapy (CPAP therapy). Only patients with genetically confirmed diagnosis of MFS (gene with causative mutation: *FBN 1*) were analyzed. Therapy and management of the MFS were carried out according to current guidelines [15–17] and according to the decision of the treating physician throughout the complete length of the observational period.

All procedures performed in studies involving human participants were in accordance with the ethical standards of the institutional and/or national research committee and with the 1964 Helsinki Declaration and its later amendments or comparable ethical standards. The Hamburg research ethics committee approved our protocol. All patients provided written informed consent. The study was investigator-initiated and without external funding. The statistical analyses and interpretation of the data were approved by all authors. The data underlying this article will be shared depending on a reasonable request to the corresponding author.

We used portable 8-channel monitoring devices for ambulatory and unattended respiratory polygraphy, which was performed at baseline. During sleep, the devices recorded nasal flow with a pressure transducer system, oxygen saturation and pulse rate by finger oximetry, body position through a magnetic sensor, snoring sounds, and thoracic and abdominal movements through belts with pneumatic cushions for pressure measurement. All patients used the device for a single, full night at home. In addition to the electronic evaluation, all polygraphy measurements were analyzed manually by a trained investigator and under the supervision of a specialist for sleep medicine. The diagnosis of sleep apnea was made in accordance with current guidelines [18, 19]. Sleep apnea was defined as an apnea–hypopnea index (AHI) > 5/h. We defined an episode of apnea as the cessation of airflow lasting ≥ 10 s and hypopnea as a decrease in airflow of ≥ 50% lasting ≥ 10 s, associated with a decrease in oxygen saturation of ≥ 4% [20].

Daytime sleepiness was assessed via Epworth Sleepiness Scale (ESS), defining daytime sleepiness with an ESS score > 10. Patients with an AHI > 5/h were referred to full polysomnography for further diagnosis and evaluation of treatment.

We performed echocardiography for left ventricular ejection fraction (LVEF) [21] and maximum aortic diameters at the level of the aortic sinuses [22]. Magnetic resonance angiography was used for diameters of the ascending and descending aortas at established levels. We did not consider diameters at aortic sites with an aortic prosthesis.

Prior results of 68 patients with Marfan syndrome have been published previously in a cross-sectional study [8]. The study was then continued with the inclusion of further patients. Medical and/or surgical treatment as well as regular follow-up was performed as recommended by guidelines. Twelve years after inclusion of the first patient (median follow-up [IQR] of 7.76 [6.84, 8.41] years), we retrospectively analyzed the patients’ electronic medical records for adverse events.

### Endpoints

The primary endpoint was cardiovascular death, and the secondary outcome was the occurrence of any aortic event. Aortic events were defined as Stanford type A dissection, Stanford type B dissection, aortic rupture, or the need for surgery or intervention for progressive dilatation of aortic aneurysm, performed according to the recommendations of the current guidelines. These are class Ic recommendations in patients who have aortic root aneurysm, with maximal aortic diameter ≥ 50 mm, or class IIa recommendations in patients with maximal ascending aortic diameters ≥ 45 mm + risk factors (family history of aortic dissection and/or aortic size increase > 3 mm/year, severe aortic or mitral regurgitation, or desire for pregnancy) [23].

Acute aortic events were defined as Stanford type A dissection, Stanford type B dissection, and aortic rupture.

Further data of interest were the diameter of the ascending and descending aorta, daytime sleepiness and laboratory values.

### Statistical analysis

The primary aim of this analysis was the impact of sleep apnea (baseline AHI) on cardiovascular mortality and aortic events of MFS patients. Demographic data, echo, laboratory data, the medical history, sleep apnea data, and Epworth Sleepiness scores (ESS) were documented in all individuals. Death, cardio-vascular death, occurrence, and type of aortic events were examined during follow-up.

Continuous data were summarized as means ± standard deviations (SD) or as medians [25th and 75th percentiles] as appropriate. Categorical data were presented as % (N). Freedom from aortic events and cardiovascular survival were estimated and graphically displayed using the Kaplan–Meier method. Survival estimates were stratified by the presence of sleep apnea and shown with 95% confidence intervals.

Effects of sleep apnea on cardiovascular mortality and acute aortic events were examined using Cox proportional hazards models. The Cox models were adjusted for age and body mass index (BMI) using restricted cubic spline functions with three knots. Results were presented with hazard ratios and 95% confidence intervals.

Median follow-up and first and third quartiles were estimated using the reverse Kaplan–Meier method.

All p-values were two-sided and a p-value < 0.05 was considered significant. All calculations were performed with the statistical analysis software R (R Core Team, 2021).

## Results

**Fig. 1:**
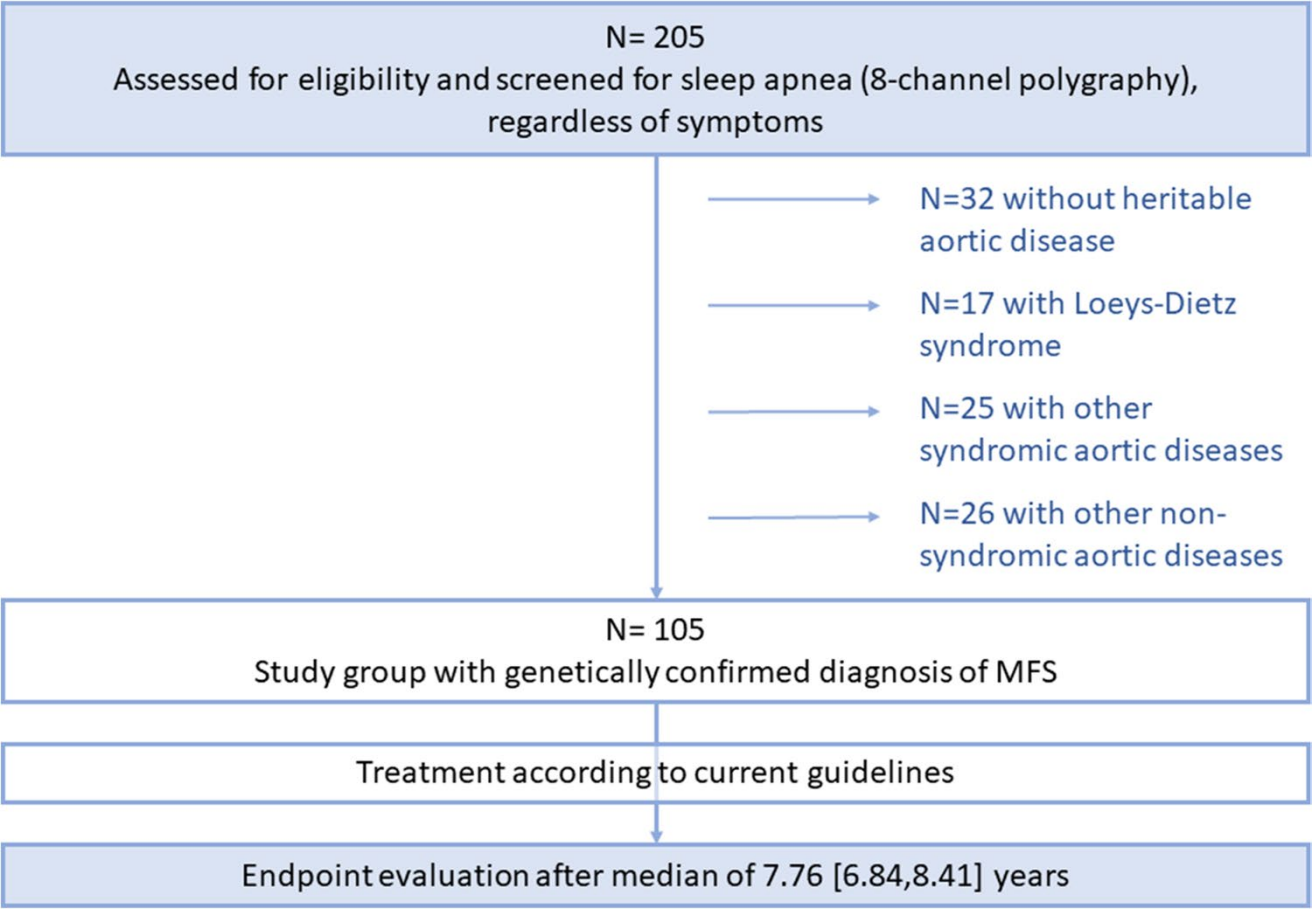
Flowchart of the study

Between February 2007 and December 2017, 205 individuals were screened for sleep apnea. We excluded 32 individuals, in whom the diagnosis of heritable thoracic aortic disease (HTAD) was not confirmed, as well as 17 patients with Loeys-Dietz syndrome, 25 patients with other syndromic aortic diseases, and 26 patients with other non-syndromic aortic diseases (Fig. 1).

Finally, the study group consisted of 105 individuals with Marfan syndrome (mean age 40 ± 13 years, 54% women). Table 1 shows the baseline characteristics of the total study cohort, including age, preexisting interventions or surgery, baseline aortic diameter, and laboratory values.

**Table 1:** Baseline data (*n* = 105)

Characteristic	*N*	Marfan syndrome *N* = 105
Age (years)	105	40 ± 13
Women	105	57 (54%)
Body mass index (kg/m^2^)	105	23.5 ± 4.9
Baseline measurements		
proBNP	103	110 [57–253]
LV-EF (%)	105	56 ± 10
LVEDD (mm)	58	55 ± 10
Ascending aorta diameter (mm) (only in patients without prior aortic root surgery)	62	37 ± 7
Ascending aorta diameter prior surgery (mm) (only in patients with prior aortic root surgery)	30	53 ± 8
Descending aorta diameter (mm)	98	26 ± 10
Pre-existing aortic events		
Prior aortic surgery	105	45 (43%)
Prior aortic root surgery	105	44 (42%)
Prior aortic event	105	
No event		59 (56%)
A dissection		17 (16%)
B dissection		5 (4.8%)
Elective intervention for progressive dilatation		23 (22%)
Abdominal aortic aneurysm		1 (1%)

For continuous variables, data are presented as the means and standard deviations. b (a-c) represent the median b with (lower quartile a and the upper quartile c) for continuous variables. For categorical variables, results are expressed as frequencies and percentages. *N* is the number of non-missing values

The mean BMI (SD) was 23.5 (± 4.9), and baseline brain natriuretic peptide (BNP) (interquartile range, IQR) was 110 (57, 253) pg/ml. Of all patients, 43% (45/105) had prior aortic surgery and preceding aortic dissection type A was present in 16% (17/105) at baseline (Table 1).

The results of sleep apnea screening are shown in Table 2. Sleep apnea with an AHI > 5/h was diagnosed in 21.0% (22/105) of patients. Mild sleep apnea was observed in 13% (14/105) of all patients and moderate to severe sleep apnea in 7.6% (8/105). CPAP therapy was initiated in 7 patients (7%).

**Table 2:** Sleep apnea data (*n* = 105)

Characteristic	*N*	Marfan syndrome *N* = 105
ESS Score	105	6.9 ± 3.8
AHI	105	5 ± 8
ODI	105	5 ± 8
Sleep apnea category	105	
No sleep apnea (AHI ≤ 5)		83 (79%)
Mild (AHI 6–15)		14 (13%)
Mod/Severe (AHI > 15)		8 (7.6%)
CPAP therapy indicated	105	7 (6.7%)
Predominantly central sleep apnea in patients with sleep apnea (> 50% of episodes + AHI > 5)	22	7 (32%)

For continuous variables, data are presented as means and standard deviations. For categorical variables, results are expressed as frequencies and percentages. *N* is the number of non-missing values

Predominately central sleep apnea was present in 32% (7/22) of patients with sleep apnea.

### Endpoints

The median follow-up (IQR) was 7.76 (6.84, 8.41) years. Five of 105 (4.8%) patients were lost to follow-up.

Death from any cause was observed in 9.5% (10/105) of all individuals with MFS. A cardiovascular cause was present in 80% (8/10) of the deceased. Further details of the causes of deaths are shown in Table 3. Aortic events occurred in 35/105 patients (33%) during follow-up (Table 3).

**Table 3:** Outcome data—death and aortic events (*n* = 105)

Characteristic	*N*	Marfan syndrome *N* = 105
Death (all cause)	105	10 (9.5%)
Cause of death	10	
Non cardiovascular		2 (20%)
Cardiovascular		8 (80%)
Cause of death (in detail)	10	
*Cardiovascular cause*		
Aortic dissection		2 (20%)
Aortic rupture		2 (20%)
Progression of heart failure		1 (10%)
Sudden cardiac death, arrhythmia		1 (10%)
Mesenteric ischemia after: a) enlargement of dissecting aneurysm of desc. aorta b) extensive surgery of aneurysm of desc. aorta		2 (20%)
*Non cardiovascular cause: sepsis*		2 (20%)
Aortic diameter		
Ascending aorta diameter (mm) at follow-up	80	35.0 ± 6.2
Descending aorta diameter (mm) at follow-up	89	28 ± 13
Aortic event (any)	105	35 (33%)
Type:	100	
A dissection		4 (4%)
B dissection		3 (3%)
Elective intervention for progressive dilatation (aortic dilatation or false lumen expansion)		29 (29%)
Location and type:		
Proximal aortic event	100	24 (24%)
Distal aortic event	100	16 (16%)

For categorical variables, results are expressed as frequencies and percentages. *N* is the number of non-missing values

**Fig. 2:**
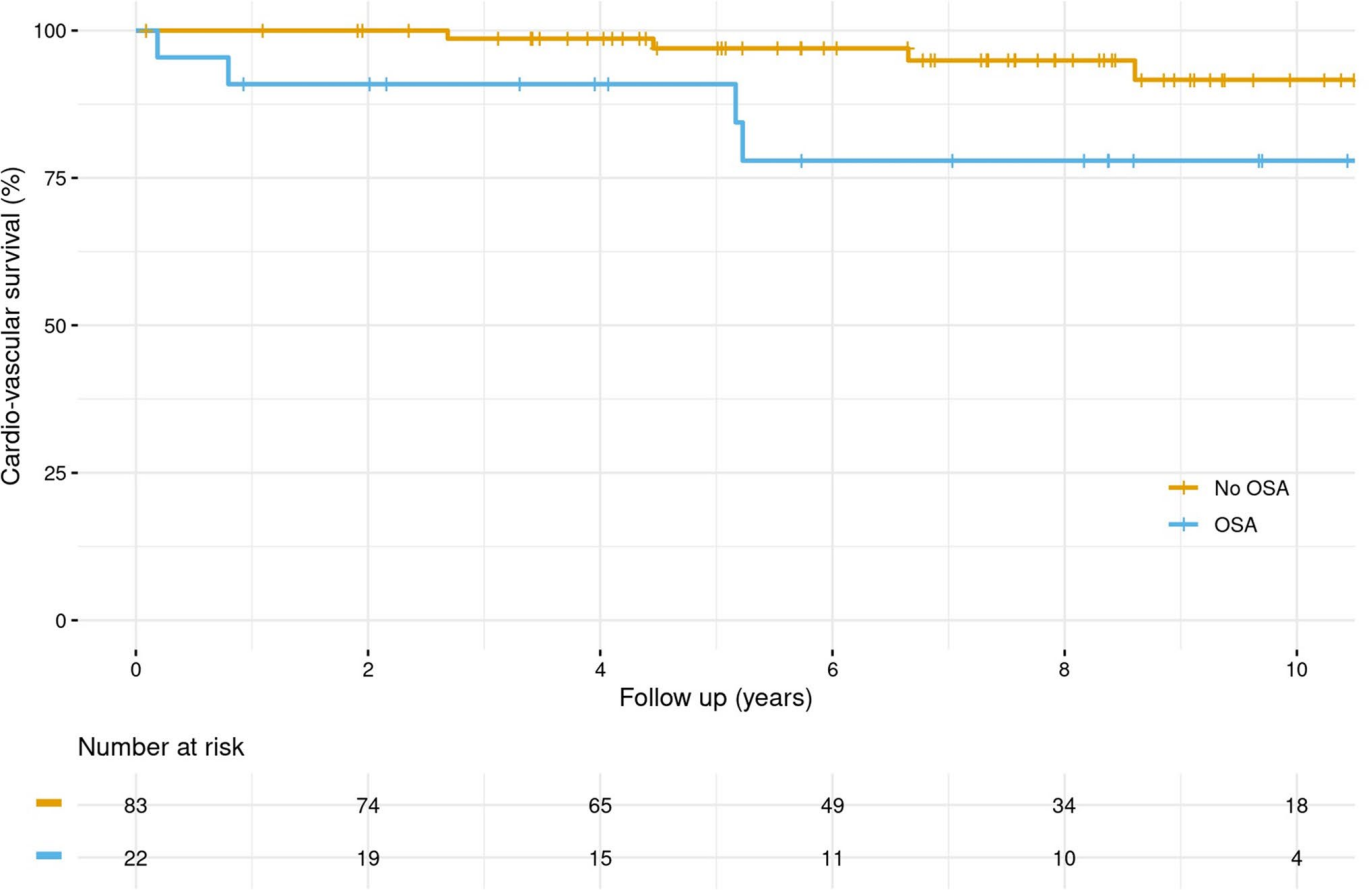
Kaplan–Meier curves for the primary outcome: **A** for MFS patients without sleep apnea (No OSA) at baseline (AHI ≤ 5); **B** for MFS patients with sleep apnea (OSA) at baseline (AHI > 5). The first primary outcome was a death from cardiovascular causes

Figure 2 shows the primary outcome of cardiovascular death in relation to sleep apnea. Individuals with sleep apnea showed a significantly lower survival rate with regard to cardiovascular death, compared to patients without sleep apnea (*p* = 0.0309). After adjusting for age and BMI, the AHI score shows to be an independent risk factor for cardiovascular death with a HR [95% CI] of 1.712 [1.061–2.761], referring to an 8-unit increase in AHI score (*p* = 0.0276). That means, in case of an increase of AHI from 5 to 15/h, there is a 22% increase in risk for cardiovascular death.

**Fig. 3:**
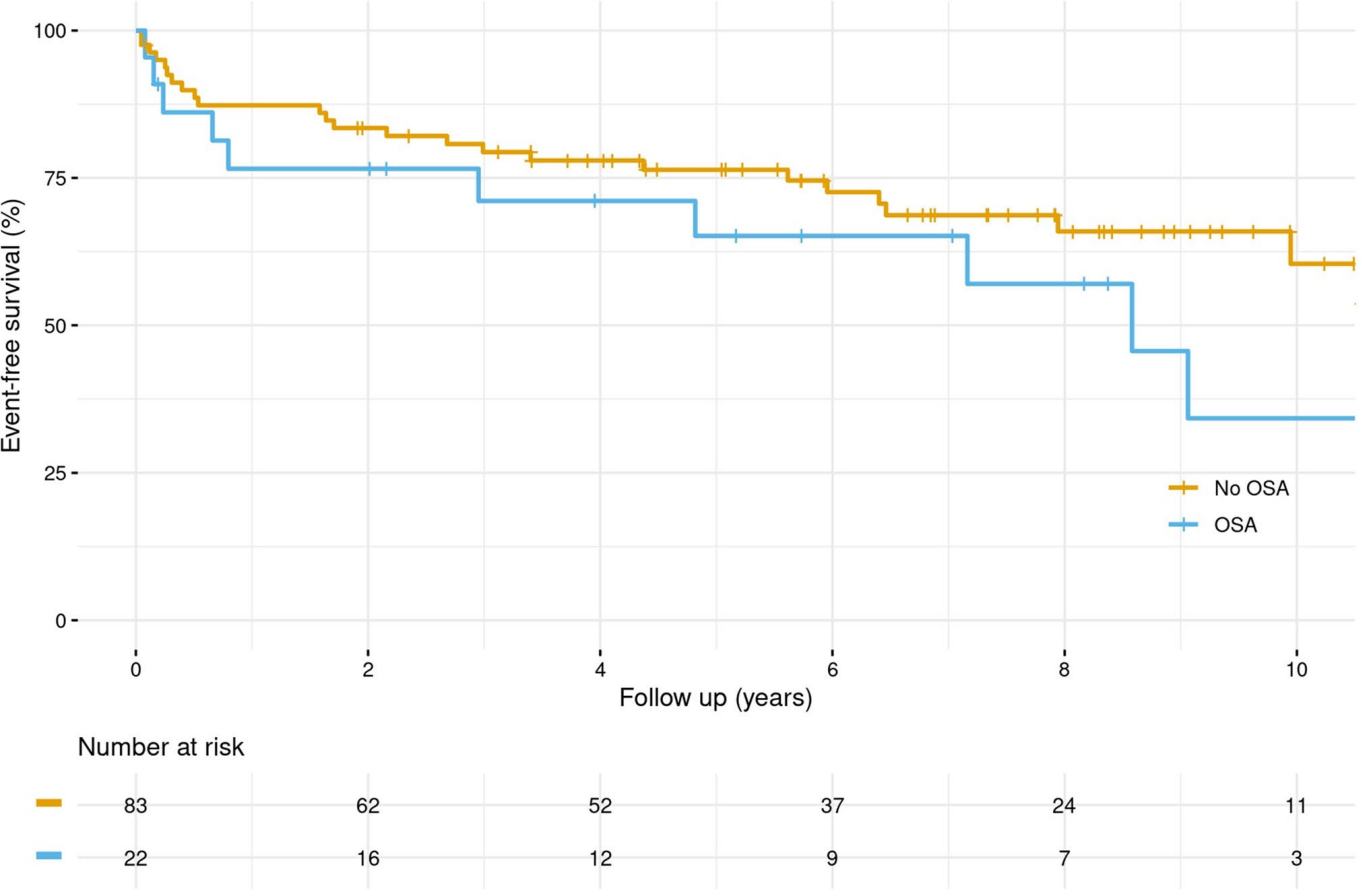
Kaplan–Meier curves for first secondary outcome: **A** for MFS patients without sleep apnea (No OSA) at baseline (AHI ≤ 5); **B** for MFS patients with sleep apnea (OSA) at baseline (AHI > 5). The first secondary outcome was the occurrence of any aortic events

Regarding the secondary outcome of aortic events, there was no significant effect of AHI score after adjusting for age and BMI (HR [95% CI]: 0.965 [0.617–1.509]), referring to an 8-unit increase in AHI score. Similar effects were observed for the AHI score on acute aortic events (HR [95% CI]: 1.247 [0.508–3.058]). Figure 3 shows that patients with sleep apnea had a lower “aortic event”-free survival compared to patients without sleep apnea, but without significant difference between the groups (*p* = 0.215). The results for acute aortic events were similar and without a significant impact (*p* = 0.216).

Additionally, the occurrence of sleep apnea or the AHI score had no significant impact on the proximal or distal aortic growth rate (*p* = 0.619 and *p* = 0.122, respectively).

An analysis of the secondary endpoint aortic events in the subgroup of patients with (45/105) and without (60/105) prior aortic surgery showed a weak effect of the AHI-score in patients without prior surgery (HR [95% CI]: 1.339 [0.878–2.044]) without reaching significance (*p* = 0.175). There was no effect in patients with prior aortic surgery (HR [95% CI]: 0.799 [0.373–1.714], *p* = 0.565).

## Discussion

### Main findings

We present an observational study of 105 Marfan patients with a median 7.76-year follow-up period from baseline. It is the first study focusing on the primary endpoint cardiovascular death in relation to sleep apnea in this rare disease. Our results demonstrate that sleep apnea is associated with an increased risk for cardiovascular death, independent of the patients’ BMI or age. There was no effect on the secondary endpoints 1) aortic events in general 2) acute aortic events, or 3) the proximal or distal aortic growth rate, probably due to the high number of patients with prior aortic surgery.

### Sleep apnea and mortality

Our study establishes an association of sleep apnea with cardiovascular mortality in Marfan syndrome. There may be several explanation for this finding.

First, sleep apnea, in general, leads to an increased risk of cardiovascular disease, including difficult-to-control blood pressure, coronary artery disease, congestive heart failure, arrhythmias, and stroke [14]. It is known that individuals with sleep apnea have a higher prevalence of atrial and ventricular arrhythmia compared to the general population [24]. Additionally, an increased risk of cardiovascular mortality was reported in individuals with severe sleep apnea [25]. The negative effects of sleep apnea on the cardiovascular system in MFS are probably similar to non-Marfan patients, and a reasonable explanation for the increased risk for cardiovascular death in Marfan patients with sleep apnea. In a cross-sectional study, Muiño‐Mosquera et al. confirmed that Marfan patients with sleep apnea tend to have higher systolic blood pressures, larger distal aortic diameters, and a higher prevalence of ventricular arrhythmias [9]. These differences were, however, not significant after adjusting for confounders [9].

Second, MFS is not only an aortic disease, but has several manifestations with possible risks for cardiovascular death, including ventricular arrhythmias and myocardial involvement [5]. Due to great achievements in medical and surgical diagnostics and therapy, death from other cardiovascular causes, excluding aortic events, have become more visible and treatable in the last years [26]. Our results are in line with these findings, showing 20% deaths of cardiovascular causes beside aortic events.

In our study, we observed high rates of mild sleep apnea, which may indicate an early state of sleep apnea in this rather young population. Yet, we were able to show that sleep apnea is a significant risk factor for cardiovascular death. Even in case of an increase of AHI from 5 to 15/h, there is a 22% increase in risk for cardiovascular death. This novel finding demonstrates the relevance of sleep apnea screening in this group of patients as an important adjunct to the management of Marfan syndrome. Based on these results, further studies are needed to evaluate different therapy options for sleep apnea in this group of patients, preferably as multicenter trials.

### Sleep apnea and aortic events

The results of our study did not show a significant effect of sleep apnea or the AHI score on aortic events or the proximal or distal aortic growth rate in patients with Marfan syndrome. Interestingly, further analyses of the subgroups of patients with and without prior aortic surgery showed that there was a weak effect of the AHI score on aortic events in patients without prior surgery. Due to the low number of patients in the subgroup, we did not reach the level of significance. In contrast to this subgroup, there was absolutely no effect in patients with prior aortic surgery. Therefore, we believe that the high number of patients with prior aortic surgery (42%) might have influenced the aortic event rate during follow-up and therefore be one explanation for the non-significant effect of the AHI score on the secondary outcome.

Limited data is published regarding this important topic. Sowho et al. showed that a high risk for sleep apnea (detected by a composite survey score) was associated with aortic enlargement and a threefold increased risk of having prior aortic root replacement in patients with Marfan syndrome [27]. Kohler et al. compared Marfan patients with and without sleep apnea and observed a significantly shorter aortic event-free survival in sleep apnea patients [13]. They showed an association between the AHI score and aortic events independent of the patients’ BMI. However, this association was no longer significant after adjusting for further covariates like age, gender, baseline aortic diameter, systolic blood pressure, and antihypertensive medication [13].

Regarding the general population beside Marfan syndrome, several studies indicate that sleep apnea alone elevates the risk for aortic dissection, aortic dilatation, and aortic rupture [28–30], whereas others did not confirm these findings [31]. It was also observed that the duration of an oxygen saturation < 90% influenced the sizes of the ascending aorta and the main pulmonary artery, showing greater dimensions in patients with sleep apnea [32].

Summing up, whether sleep apnea leads to an increased risk for aortic events in MFS remains unclear and needs further investigation in larger cohorts, preferably in patients without prior aortic surgery or intervention.

## Conclusions and expert recommendations in framework of predictive, preventive and personalized medicine

The PPPM proposes, implements, and supports the need of a paradigm shift from reactive medical services to predictive, preventive, and personalized medicine concepts of health [33, 34]. Care of individuals with Marfan syndrome aims to prevent patients from life threatening complications. However, current prevention is of a secondary (detection of aortic dilatation) and tertiary (after aortic dissection) nature [1]. In order to enforce the paradigm shift, primary care strategies need to be encouraged in order to offer a better and more individualized concept and approach to the patient.

Sleep apnea, a modifiable risk factor with sufficient treatment options [22, 35], is emerging as an independent predictor of cardiovascular death in MFS. As seen in this study, even with mild sleep apnea alone, the impact on mortality was significant. Hence, it seems mandatory to screen all individuals with MFS for sleep apnea. We recommend using ambulatory devices first with confirmation at sleep laboratory in those individuals with an AHI > 5/h.

Treatment of sleep apnea may target primary prevention regarding cardiovascular death. Nevertheless, there are different therapy options for sleep apnea [22, 35]. While positive airway pressure (PAP) therapy is recommended for patients with obstructive sleep apnea [35], adaptive servo-ventilation increased all-cause and cardiovascular in patients with central sleep apnea and heart failure [36].

Therefore, we recommend to evaluate the different therapy options in future studies, to develop a personalized treatment protocol tailored to type and severity of sleep apnea, comorbidities, and observed treatment effects. Individuals, with both MFS and sleep apnea, should be included to evaluate the impact of the preventive measures with regard to cardiovascular death.

## Limitations

Few aspects may weaken reproducibility in other populations. First, our study was observational with varying observation times across individuals. Second, besides age and BMI, other confounders may exist. Third, sleep apnea screening was offered to all patients, regardless of symptoms. Nevertheless, several patients did not participate, which may have excluded an important proportion of patients leading to selection bias of the study cohort. Additionally, the power of the comparisons was low due to the small number of patients and the low number of events. The effects on the secondary endpoints might have been clearer in a larger group of patients. However, the effect of sleep apnea on the primary endpoint cardiovascular mortality was seen clearly.

## Supplementary Information

Below is the link to the electronic supplementary material.

Supplementary file1 (DOCX 18 KB)

## Data Availability

The datasets generated during and/or analyzed during the current study are available from the corresponding author on reasonable request.

## Abbreviations

AHI: Apnea-hypopnea index
BMI: Body mass index
CPAP: Continuous positive airway pressure therapy (CPAP therapy)
ESS: Epworth Sleepiness Scale
HR: Hazard ratio
HTAD: Heritable thoracic aortic diseases
IQR: Interquartile range
LVEF: Left ventricular ejection fraction
MFS: Marfan syndrome
ODI: Oxygen desaturation index
PAP: Positive airway pressure therapy (PAP therapy)
PPPM: Predictive, preventive, and personalized medicine
SD: Standard deviations
