# Supplementary material for: Sleep apnea predicts cardiovascular death in patients with Marfan syndrome: a cohort study
Source: EPMA J. 2022 Jul 29;13(3):451–60. doi: 10.1007/s13167-022-00291-4 (PMC9437159; doi:10.1007/s13167-022-00291-4)
Supplement: Supplementary file 1 — Supplementary file1 (DOCX 18 KB) [file 13167_2022_291_MOESM1_ESM.docx]

**Supplementary files:** Gessler et al. Sleep apnea predicts cardiovascular death in patients with Marfan syndrome

**S1: Statistical References**

1. Allaire, JJ, Yihui Xie, Jonathan McPherson, Javier Luraschi, Kevin Ushey, Aron Atkins, Hadley Wickham, Joe Cheng, Winston Chang, and Richard Iannone. 2021. Rmarkdown: Dynamic Documents for r. https://github.com/rstudio/rmarkdown.
2. Francois, Romain. 2020. Bibtex: Bibtex Parser. https://CRAN.R-project.org/package=bibtex.
3. Kassambara, Alboukadel, Marcin Kosinski, and Przemyslaw Biecek. 2021. Survminer: Drawing Survival Curves Using ’Ggplot2’. https://CRAN.R-project.org/package=survminer.
4. R Core Team. 2021. R: A Language and Environment for Statistical Computing. Vienna, Austria: R Foundation for Statistical Computing. https://www.R-project.org/.
5. Sjoberg, Daniel D., Michael Curry, Margie Hannum, Joseph Larmarange, Karissa Whiting, and Emily C. Zabor. 2021. Gtsummary: Presentation-Ready Data Summary and Analytic Result Tables. https://CRAN.R-project.org/package=gtsummary.
6. Terry M. Therneau, and Patricia M. Grambsch. 2000. Modeling Survival Data: Extending the Cox Model. New York: Springer.
7. Therneau, Terry M. 2021. A Package for Survival Analysis in r. https://CRAN.R-project.org/package=survival.
8. Wickham, Hadley. 2016. Ggplot2: Elegant Graphics for Data Analysis. Springer-Verlag New York. https://ggplot2.tidyverse.org.
9. Wickham, Hadley, Mara Averick, Jennifer Bryan, Winston Chang, Lucy D’Agostino McGowan, Romain François, Garrett Grolemund, et al. 2019. “Welcome to the tidyverse.” Journal of Open Source Software 4 (43): 1686. https://doi.org/10.21105/joss.01686.
10. Xie, Yihui, J. J. Allaire, and Garrett Grolemund. 2018. R Markdown: The Definitive Guide. Boca Raton, Florida: Chapman; Hall/CRC. https://bookdown.org/yihui/rmarkdown.
11. Xie, Yihui, Christophe Dervieux, and Emily Riederer. 2020. R Markdown Cookbook. Boca Raton, Florida: Chapman; Hall/CRC. https://bookdown.org/yihui/rmarkdown-cookbook.
